# Supplementary material for: Codon Usage Bias Analysis in Macronuclear Genomes of Ciliated Protozoa
Source: Microorganisms. 2023 Jul 18;11(7):1833. doi: 10.3390/microorganisms11071833 (PMC10384029; doi:10.3390/microorganisms11071833)
Supplement: Supplementary file 1 [file microorganisms-11-01833-s001.zip › Supplementary Material.pdf]

Supplementary Material

Microoriganisms

Codon Usage Bias Analysis in Macronuclear Genomes of Ciliated Protozoa

Yu Fu [1], Fasheng Liang [1], Congjun Li [1], Alan Warren [2], Mann Kyoon Shin [3] and Lifang Li [1,*]

[1] Laboratory of Marine Protozoan Biodiversity and Evolution, Marine College, Shandong University, Weihai 264209, China; fy_sdu@163.com (Y.F.); lfsdante@163.com (F.L.); licongjun123june@163.com (C.L.)

[2] Department of Life Sciences, Natural History Museum, London SW7 5BD, UK; a.warren@nhm.ac.uk

[3] Department of Biology, University of Ulsan, Ulsan 44610, Republic of Korea; mkshin@ulsan.ac.kr

* Correspondence: qd_liliy@sina.com or lifangli@sdu.edu.cn

Supplementary Tables

Table S1. GenBank accession numbers of the 21 ciliate species.

Table S2. The correlation analysis of the 21 ciliate species.

Table S3. The ENc radio of the 21 ciliate species.

Table S4. The RSCU value of the 21 ciliate species.

Table S5. The putative optimal codons of the 21 ciliate species.

Table S6. The third position codon bias of the 21 ciliate species.

Supplementary Figures

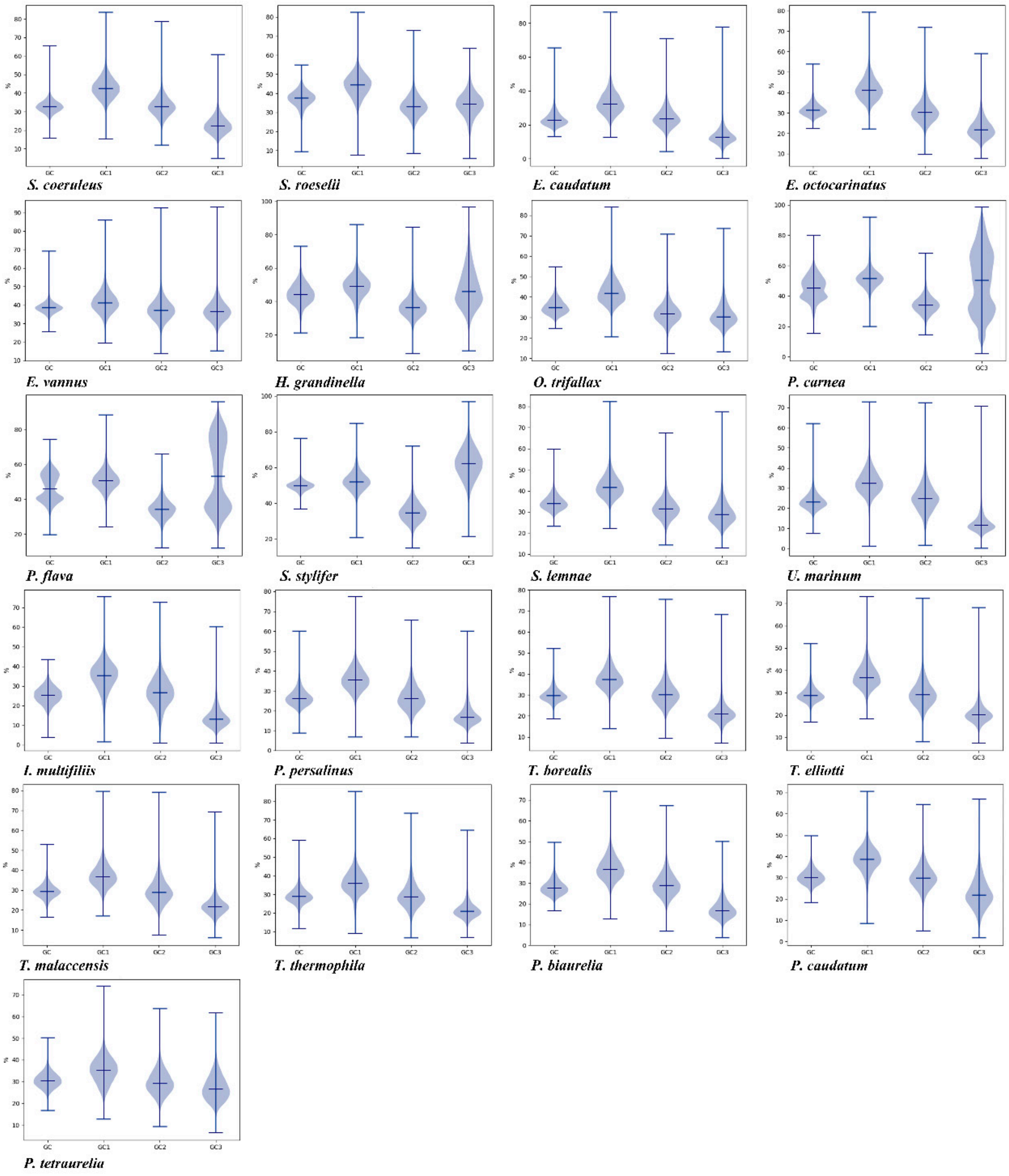

Supplementary Figure S1. Normal box plots for the range of GC, GC1, GC2, and GC3 content in the 21 ciliate species showing the probability density of data at different values.

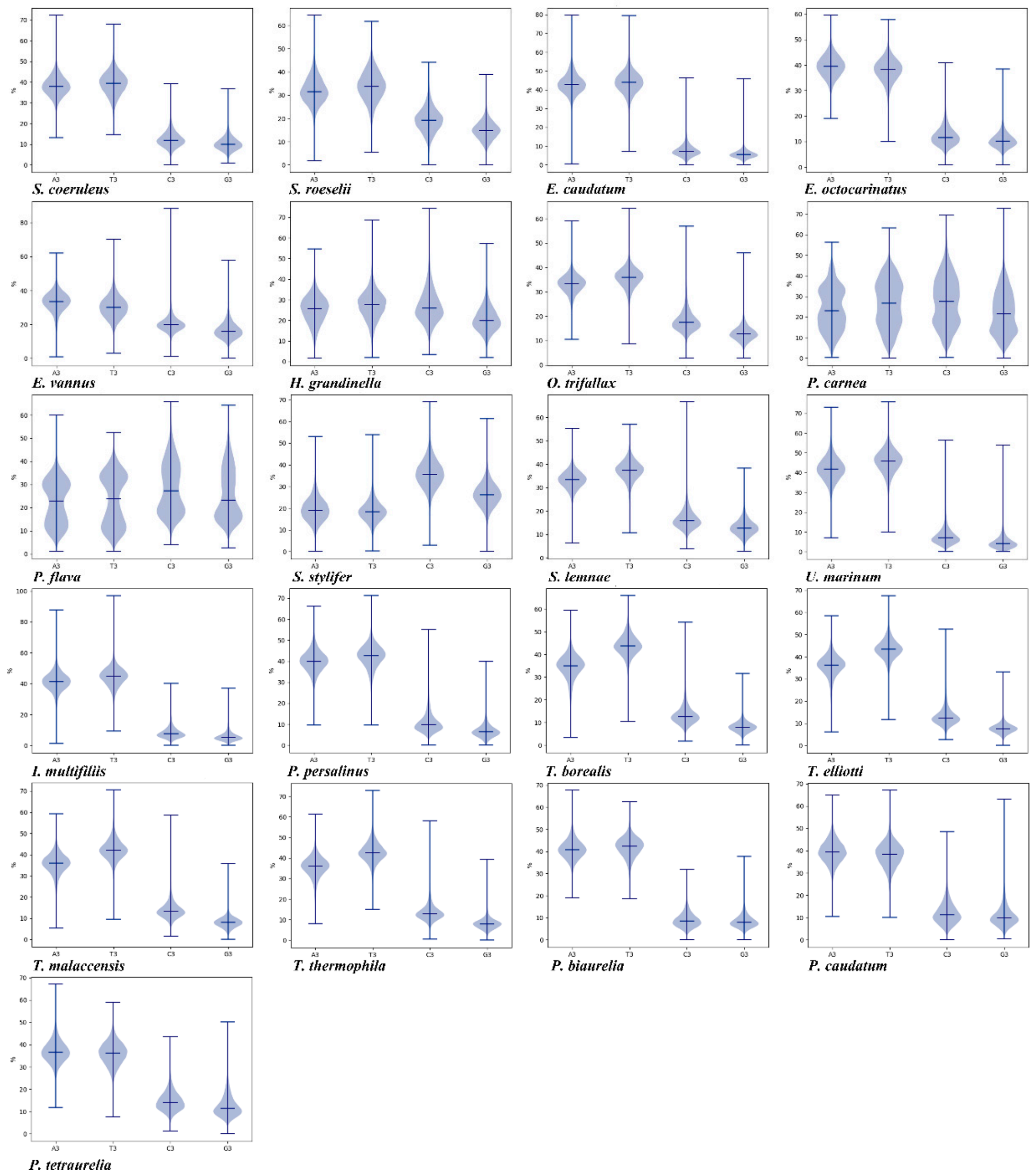

Supplementary Figure S2 Normal box plots for the range of A3, T3, C3 and, G3 content in the 21 ciliate species showing the probability density of data at different values.

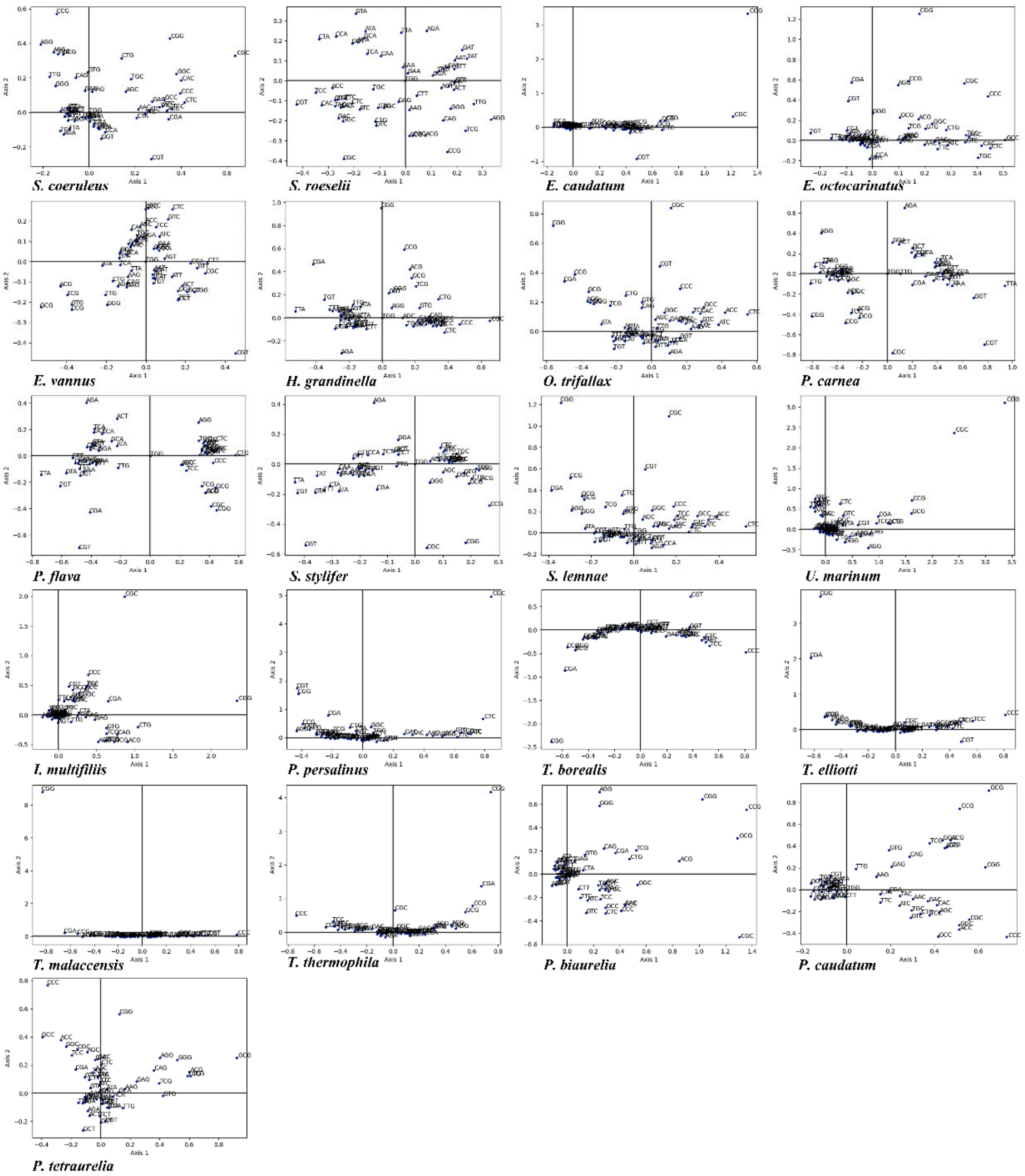

Supplementary Figure S3. Codon correspondence analysis plot of each gene in the 21 ciliate species. Axis 1 and axis 2 represent the largest contributors to the CUB values of genes. The black dots represent the codons.
